# Efficacy of cabazitaxel and androgen splicing variant-7 status in circulating tumor cells in Asian patients with metastatic castration-resistant prostate cancer

**DOI:** 10.1038/s41598-022-22854-1

**Published:** 2022-10-26

**Authors:** Takeshi Ashizawa, Masayoshi Nagata, So Nakamura, Hisashi Hirano, Naoya Nagaya, Yan Lu, Shigeo Horie

**Affiliations:** 1 Department of Urology, Juntendo University Graduate School of Medicine, 2-1-1 Hongo Bunkyo-Ku, Tokyo, 1138431 Japan; 2 Department of Advanced Informatics of Genetic Diseases, Juntendo University Graduate School of Medicine, Tokyo, Japan

**Keywords:** Oncology, Urology

## Abstract

Androgen receptor splice variant-7 (AR-V7) expression in circulating tumor cells (CTCs) in metastatic castration-resistant prostate cancer (mCRPC) is associated with abiraterone and enzalutamide resistance. We determine whether cabazitaxel (CBZ) is equally effective in AR-V7-positive and -negative CRPC and whether AR-V7-positive patients retain CBZ sensitivity. This is the first prospective, open-label, Asian validation study of CBZ in Japanese patients with mCRPC after docetaxel (n = 48; four CBZ cycles; 2017–2020, Juntendo University Hospitals). Primary endpoint was prostate-specific antigen response rate (PSA-RR); secondary endpoints included overall survival (OS), bone scan index (BSI) PSA-RR (≥ 50% decline from baseline) for CTC−/ARV7−, CTC+ /ARV7−, and CTC +/ARV7+ groups. PSA-RR ≥ − 30% was 38% (18/48) and ≥ − 50% was 26% (12/48). BSI-change rate ≥ − 30% was 19% (9/41) and ≥ − 50% was 17% (8/41). Median OS was 13.7(12.2–18.9) months. PSA decline in early CBZ treatment associated with OS (*p* = 0.00173). BSI decline associated with OS (*p* = 0.0194). PSA-RR(≥ 50%) was 43%(6/14) in CTC−/ARV7−, 19%(5/26) in CTC+ ARV7−, and 12%(1/8) in CTC+/ARV7+ (* p* > 0.05). AR-V7 in CTCs at baseline not associated with OS. AR-V7 was not associated with CBZ resistance in CTCs. Reductions in BSI and PSA in early stages of CBZ treatment may predict OS.

## Introduction

Androgen receptor (AR) signaling is an important survival pathway for castration-resistant prostate cancer (CRPC) cells. In most CRPC cells, the AR maintains its function as a nuclear receptor in a ligand-independent manner, allowing it to evade androgen deprivation therapy^1^. Approximately 20–40% of the patients with CRPC have primary resistance to new anti-androgen agents such as enzalutamide and abiraterone^2,3^. Moreover, virtually all patients who initially respond to these agents develop secondary resistance over time. One plausible explanation for this resistance could be the presence of AR splice variants^4,5^. Although truncated AR in these splice variants is incapable of binding ligand, it is constitutively active as a transcription factor. One of the variants by which the AR can become ligand independent is the androgen receptor splice variant-7 (AR-V7). AR-V7 lacks a ligand-binding domain and remains functionally active even in the absence of androgen, which can lead to a poor prognosis^6,7^. AR-V7 expression in circulating tumor cells (CTCs) from patients with CRPC has recently been linked to resistance to abiraterone and enzalutamide^4,5^.

Theoretically, taxane-based chemotherapy should be effective against CRPC with constitutively active AR because its anti-tumor effect is exerted through a different mechanism, as a tubulin-stabilizing agent. Antonarakis et al. reported that the detection of AR-V7 in CTCs from men with metastatic CRPC (mCRPC) was not associated with primary resistance to taxane chemotherapy^8^. In addition, Onstenk et al. reported that cabazitaxel (CBZ) response may be independent of the AR-V7 status of CTCs from patients with mCRPC, and that CBZ could be a viable treatment option for patients with AR-V7-positive CTCs^9^. However, there are limited data on the efficacy of taxane-based chemotherapy for AR-V7-positive CRPC.

This prospective, real-world study aims to investigate if CBZ is equally effective in AR-V7-positive and AR-V7-negative CRPC, and if AR-V7-positive patients retain CBZ sensitivity. This first-of-its-kind Asian validation study is an open-label, single-arm, single-center (Juntendo University hospital) cohort study to assess the efficacy of CBZ in Japanese patients with mCRPC with or without AR-V7-positive CTCs who have demonstrated disease progression on docetaxel treatment*.*

## Results

A total of 55 patients with mCRPC were enrolled and received CBZ, with the majority of patients (n = 48) receiving more than four cycles of CBZ. Table 1 shows patient characteristics at baseline; the median (range) age was 71 (51–82) years, and the median time to CRPC diagnosis was 10 (3–78) months. At the time of diagnosis, 83% (40/48) of the patients had locally advanced prostate cancer, and 79% (38/48) of the patients had high-grade prostate cancer (Gleason score was ≥ 8). As local treatment, 13% (6/48) of the patients had surgery (radical prostatectomy or robotic laparoscopic prostatectomy), and 27% (13/48) of the patients had radiation therapy (intensity modulated radiation therapy [IMRT] for primary cancer or irradiation to bone metastasis). Prior hormonal or chemotherapy treatment lines for CRPC prior to CBZ treatment initiation included: 19% of the patients (n = 9) received one line of treatment, 39% (n = 17) received two lines of treatment, 26% (n = 13) received three lines of treatment, and 19% (n = 9) received ≥ 4 lines of treatment.

**Table 1:** Patient characteristics at the time of prostate cancer diagnosis and mCRPC .

Patient characteristics	All patients (n = 48)
Age, median (range), y	71 (51–82)
Time to CRPC, median (range), m	10 (3–78)
**Tumor stage at diagnosis, No. (%)**	
T1/T2	8 (17)
T3/T4	40 (83)
**Gleason sum at diagnosis, No. (%)**	
< 8	10 (21)
≥ 8	38 (79)
**Type of local treatment, No. (%)**	
Surgery	6 (13)
Radiation therapy (primary or metastasis)	13 (27)
None	29 (60)
**No. of prior hormonal or chemotherapies for**	
CRPC, median (range)	2 (1–6)

mCRPC, metastatic castration-resistant prostate cancer; m, months; no, number; PSA, prostate specific antigen; y, years.

Initial treatments for mCRPC included: enzalutamide, abiraterone, radium-223 chloride, flutamide, ethinylestradiol, estramustine phosphate, and docetaxel. Prior to CBZ treatment, all patients had received docetaxel; the median (range) was 6 (1–39). Prior to enrollment, 17 patients (35%) had received abiraterone, 30 patients (62%) had received enzalutamide, and 10 patients (21%) had received both enzalutamide and abiraterone (Table 2).

**Table 2:** Baseline characteristics of patients with mCRPC before cabazitaxel treatment.

Baseline characteristics	All patients, n = 48, Number(%)
**ECOG-PS**	
0–1	35 (73)
2–3	13 (27)
Serious comorbidity*	18 (38)
**Prior use of bicalutamide, No. (%)**	
Yes	48 (100)
No	0 (0)
**Prior use of flutamide, No. (%)**	
Yes	15 (31)
No	33 (69)
**Prior use of estramutine phosphate, No. (%)**	
Yes	8 (17)
No	40 (83)
**Prior use of ethinylestradiol, No. (%)**	
Yes	5 (10)
No	43 (90)
**Prior use of radium-223 chloride, No. (%)**	
Yes	7 (15)
No	41 (85)
**Prior use of abiraterone, No. (%)**	
Yes	17 (35)
No	31 (65)
**Prior use of enzalutamide, No. (%)**	
Yes	30 (62)
No	18 (38)
**Prior use of docetaxel, No.(%)**	
Yes	48 (100)
No	0 (0)
**Presence of bone metastases, No. (%)**	
Yes	44 (91)
No	4 (9)
**No. of bone metastases, No. (%)**	
< 6	20 (41)
≥ 6	28 (59)
**Presence of visceral metastases, No. (%)**	
Yes	13 (27)
No	35 (73)
Baseline PSA level, median (range), ng/ml	50.3 (0.1–1549)
Baseline alkaline phosphatase level, U/L	286 (114–1486)

*Include diabetes, post-myocardial infarction, post-cerebrovascular accident, liver injury, pulmonary obstructive disorder, arteriosclerosis obliterans, and other active cancers.

**Figure 1:**
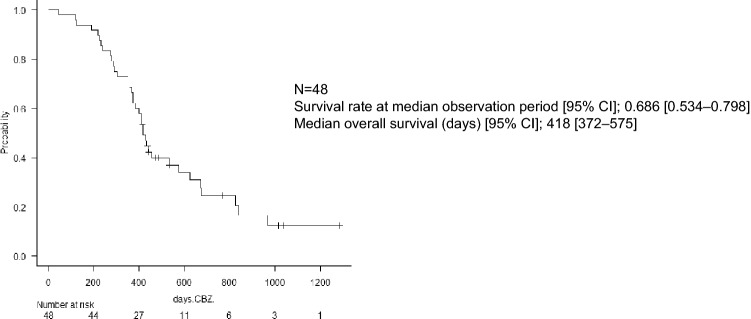
Kaplan–Meier curve of overall survival (OS) rate of all patients with CRPC after four or more cycles of cabazitaxel treatment. Survival rate at median observation period [95% CI]; 0.686 [0.534–0.798]. Median overall survival (days) [95% CI]; 418 [372–575], N = 48. CBZ, cabazitaxel; CI, confidence interval; CRPC, castration-resistant prostate cancer; OS, overall survival.

The median observation period for all 48 patients treated with CBZ was 13.7 (7.3–34.6) months. The median OS for all patients was 13.7 months (95% CI, 12.2–18.9) (Fig. 1). There was no significant difference in the number of treatment lines before CBZ treatment and the OS rate (HR, 0.98 [95% CI, 0.41–2.34]; *p* = 0.19) and no significant difference in time to CRPC and the OS (HR, 0.84 [95% CI, 0.37–1.93]; *p* = 0.85).

At baseline, CTCs were detected in 34 of 48 blood samples (70.8%), and eight of these 34 samples (16%) had detectable AR-V7-positive cells (Table 3). Seven of the eight (88%) AR-V7 positive patients had previously received either enzalutamide or abiraterone, and two of these seven (29%) AR-V7-positive patients had previously received both enzalutamide and abiraterone.

**Table 3:** Clinical characteristics of patients in the CTC-negative and CTC-positive groups.

Baseline Characteristics	CTC(−), AR-V7 (−) (n = 14)	CTC ( +), AR-V7 (−) (n = 26)	AR-V7 ( +) (n = 8)	*P* value
Age, median (range), y	71.5 (57–78)	71 (51–79)	71.5 (60–82)	0.990
Time to CRPC, median (range), m	9 (3–53)	10 (3–78)	8 (4–24)	0.710
**Tumor stage at diagnosis, No**				
T1/T2	3	3	0	0.343
T3/T4	11	23	8	
**Gleason sum at diagnosis, No**				
< 8	4	6	0	0.268
≥ 8	10	20	8	
**Type of local treatment, No**				
Surgery	3	4	1	0.966
Radiation therapy	3	7	2	
None	8	15	5	
No. of prior hormonal or chemotherapies for CRPC, median (range)	2 (1–6)	2 (1–6)	3 (1–6)	0.40
**Prior use of abiraterone, No**				
Yes	4	9	4	0.190
No	10	17	4	
**Prior use of enzalutamide, No**				
Yes	7	18	5	0.495
No	7	8	3	
**Prior use of docetaxel, No**				
Yes	14	26	8	NA
No	0	0	0	
**Presence of bone metastases, No**				
Yes	13	22	8	0.758
No	1	4	0	
**No. of bone metastases, No**				
< 6	11	16	2	0.023
≥ 6	3	10	6	
**Presence of visceral metastases, No**				
Yes	4	7	2	0.984
No	10	19	6	
Baseline PSA level, median (range), ng/ml	13.4 (0.1–181)	127 (4.89–968)	81.2 (3.24–1549)	0.011
Baseline alkaline phosphatase level, median (range), U/L	220 (114–579)	304 (136–1486)	434 (153–686)	0.091

The CTC-negative and AR-V7-negative groups included 14 patients each. The CTC-positive group included AR-V7-negative (n = 26), and AR-V7-positive (n = 8) patients.

The Kruskal–Wallis test was used for statistical analysis of patient characteristics.

*CRPC* castration-resistant prostate cancer, *m* months, *no* number, *PSA* prostate specific antigen, *y* years, (*−*) negative, (+) positive.

**Figure 2:**
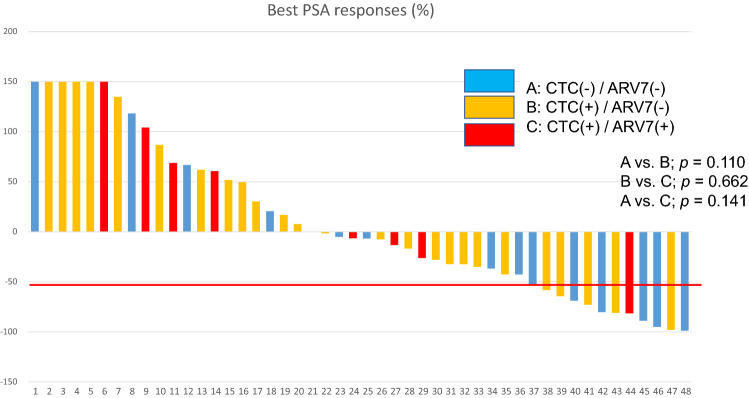
Waterfall plot of the best PSA change rate after four or more cycles of cabazitaxel. Group A, CTC (−) / AR-V7 (−); Group B, CTC ( +) / AR-V7 (−); Group C, CTC ( +) / AR-V7 ( +). A vs. B, *p* = 0.318; B vs. C, *p* = 0.391; A vs. C, *p* = 0.685. AR-V7, androgen splicing variant-7; CBZ, cabazitaxel; CRPC, castration-resistant prostate cancer; CTC, circulating tumor cell; PSA, prostate specific antigen; (−), negative; ( +), positive.

After a minimum of four cycles of CBZ treatment, the best PSA reduction rate was ≥ 30% in 18 patients (38%) and ≥ 50% in 12 patients (25%) (Fig. 2). There was no significant difference in PSA best change rate between the CTC-negative/AR-V7-positive, CTC-positive/AR-V7-negative, and CTC-positive/AR-V7-positive groups. PSA-RR (≥ 50%) was observed in 43% (6/14) of the patients in the CTC-negative/AR-V7-negative group, 29% (5/26) of patients in the CTC-positive/AR-V7-negative group, and 13% (1/8) of the patients in the CTC-positive/AR-V7-positive group. There was no significant difference between the AR-V7-positive and AR-V7-negative groups (*p* = 0.662) (Fig. 2).

Despite receiving more than three cycles of CBZ, 42% (20/48) of the patients did not show a PSA-lowering response to chemotherapy. Overall survival was significantly worse in patients who did not have a PSA-lowering response in the early stages of CBZ treatment (HR, 2.84 [95% CI, 1.22–6.57]; *p* = 0.00173) (Supplemental Figure). Therefore, the PSA decline in the early stages of CBZ treatment could predict better survival with CBZ treatment.

**Figure 3:**
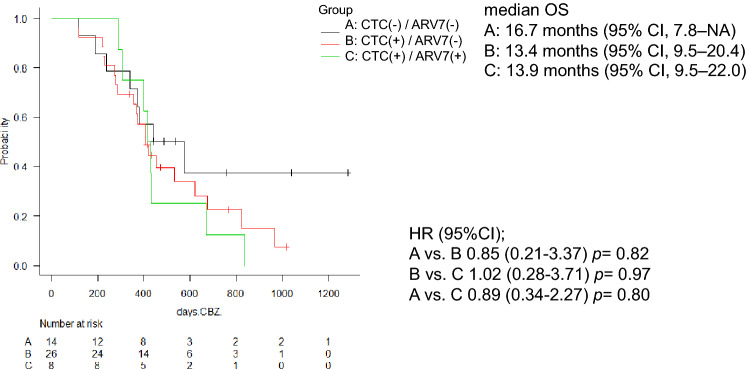
Kaplan–Meier curve of overall survival (OS) rate of groups with or without CTC and AR-V7 status at baseline. Group A, CTC (−) / AR-V7 (−); Group B, CTC ( +) / AR-V7 (−); Group C, CTC ( +) / AR-V7 ( +), analyzed by log-rank test (N = 48). Median OS was 13.9 months (95% CI, 9.5–22.0) in the group C, 13.4 months (95% CI, 9.5–20.4) in the group B, and 16.7 months (95% CI, 7.8–NA) in the group A. HR (95% CI): A vs. B, 0.85 (0.21–3.37), *p* = 0.82; B vs. C, 1.02 (0.28–3.71), *p* = 0.97; A vs. C, 0.89 (0.34–2.27) *p* = 0.80. AR-V7, androgen splicing variant-7; CI, confidence interval; HR, hazard ratio; CTC, circulating tumor cell; OS, overall survival; (−), negative; ( +), positive.

Findings from the CTC analyses showed that median OS was 13.9 months (95% CI, 9.5–22.0) in the AR-V7-positive group, 13.4 months (95% CI, 9.5–20.4) in the CTC-positive/AR-V7-negative group, and 16.7 months (95% CI, 7.8–NA) in the CTC-negative/AR-V7-negative group (Fig. 3). The presence of AR-V7 in CTCs at baseline was not associated with OS (CTC-positive/AR-V7-positive versus CTC-positive/AR-V7-negative, HR: 1.02 [95% CI, 0.28–3.71]; *p* = 0.97; CTC-positive/AR-V7-positive versus CTC-negative/AR-V7-negative, HR: 0.89 [95% CI 0.34–2.27]; *p* = 0.80).

**Figure 4:**
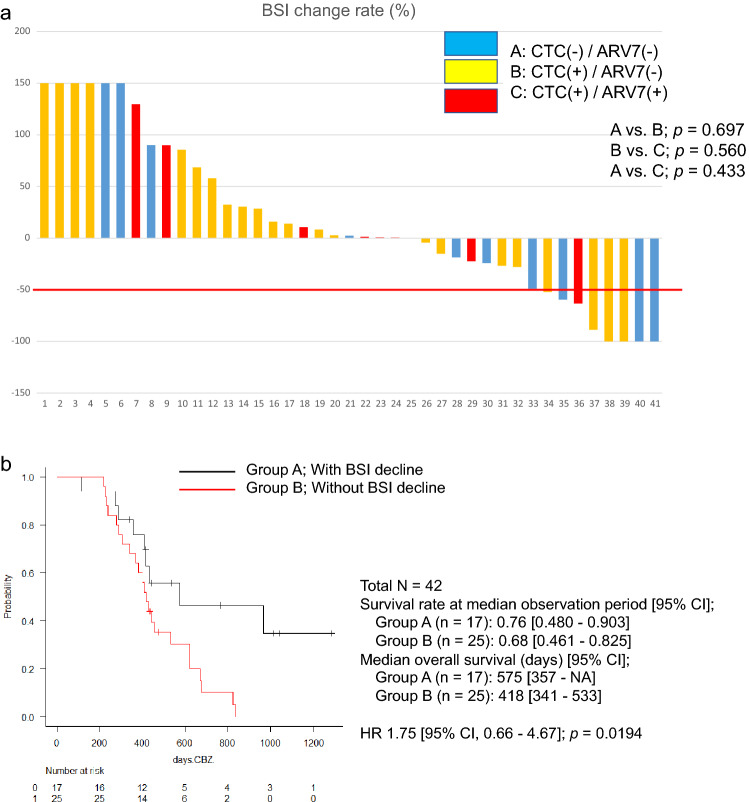
**a** Waterfall plots of percent change in bone scan index (BSI) after cabazitaxel treatment. Group A, CTC (−) / AR-V7 (−); Group B, CTC ( +) / AR-V7 (−); Group C, CTC ( +) / AR-V7 ( +) (N = 41). A vs. B, *p* = 0.697; B vs. C, *p* = 0.560; A vs. C, *p* = 0.433. **b** Kaplan–Meier curve of overall survival (OS) rate of patients with CRPC after cabazitaxel treatment, with or without bone scan index (BSI) reduction response. Group A, patients with BSI decline; Group B, patients without BSI decline (log-rank test) (N = 42). Survival rate at median observation period [95% CI]: Group A (n = 17), 0.76 [0.480–0.903]; Group B (n = 25), 0.68 [0.461–0.825]. Median overall survival (days) [95% CI]: Group A (n = 17), 575 [357–NA]; Group B (n = 25), 418 [341–533]. HR 1.75 [95% CI, 0.66–4.67], *p* = 0.0194. AR-V7, androgen splicing variant-7; BSI, Bone Scan Index; CI, confidence interval; CRPC, castration-resistant prostate cancer; CTC, circulating tumor cell; HR, hazard ratio; OS, overall survival; (−), negative; ( +), positive.

During CBZ treatment, BSI change rates were available for 41 patients; nine patients (19%) achieved a ≥ 30% reduction, and eight patients (17%) achieved a ≥ 50% reduction after 12 weeks of CBZ treatment (Fig. 4a). CTC analysis revealed that there was no significant difference in the BSI change rate between the CTC-negative, CTC-positive/AR-V7-negative, and CTC-positive /AR-V7-positive groups. However, OS was significantly worse in patients who did not have a BSI decreasing response after CBZ treatment (HR, 1.75 [95% CI, 0.66–4.67]; *p* = 0.0194). (Fig. 4b). Therefore, the effect of CBZ treatment on bone metastases, as objectively evaluated by bone scintigraphy BSI, could predict favorable survival during CBZ treatment.

For adverse events of grade 2–3 and above, CBZ administration was continued with appropriate dose reductions. Although febrile neutropenia (FN) of G3 or higher developed and the dose of CBZ was reduced in six cases, pegfilgrastim prevented severe FN and treatment-related death in the majority of patients. Although CBZ administration was not discontinued, blood transfusions were required in four patients with G3 anemia and one patient with G3 thrombocytopenia (Supplemental Table). Four patients withdrew in less than three cycles due to factors such as distance to the hospital, pain from bone metastases, and a desire to transfer to a local referral hospital, and for three patients, CBZ was discontinued before the completion of four cycles due to other disease causes (cerebral hemorrhage, myocardial infarction, and other advanced cancers). As a result, seven of the 55 patients who entered the study withdrew after receiving less than three cycles of CBZ.

## Discussion

This study was conducted in Japanese patients with mCRPC in a setting similar to the CABARESC study by *Onstenk *et al*.*, i.e., all patients had previously received docetaxel chemotherapy, and the effect of CBZ treatment was evaluated based on CTC status^10^. The median OS rate in this real-world study, which included patients with poor performance status or severe comorbidities (Table 2), was 13.7 months, which was comparable to the median OS of 15.1 months in the multinational phase III TROPIC trial^11^. The median OS in this study was also comparable to that of the other real-world clinical trials of CBZ in Japanese patients with mCRPC (10.6–16.3 months)^12–14^.

This study is the first to prospectively investigate the correlation between the presence or absence of CTCs and AR-V7 status to determine the effect of CBZ treatment in Japanese patients with mCRPC. Recent studies have reported that the presence of AR-V7 in CTC is associated with resistance to the novel AR-targeted therapies, such as abiraterone or enzalutamide, but not to taxane-based chemotherapy^4,8^. In this study, we found that the efficacy of CBZ was independent of AR-V7 status in both patients who were CTC-positive and -negative (Fig. 2). These findings are comparable to those from the PROPHECY and CABARESC studies, where the response to CBZ appeared to be independent of the AR-V7 status of CTCs from patients with mCRPC^10,15^. Moreover, there were no significant differences in OS rates from the start of CBZ chemotherapy for the three groups: AR-V7-positive, AR-V7-negative/CTC-positive, and CTC-negative (Fig. 3). This suggests that CBZ should be used preferentially in patients with mCRPC with AR-V7-positive CTCs after docetaxel treatment. Markowski et al*.* reported a similar viewpoint for AR-V7-positive patients, where treatment was frequently changed from AR targeted therapy to taxane-based chemotherapy (43%), or inclusion into clinical trials (43%)^16^. Zhang et al. reported that AR-V7-positive patients with mCRPC benefit from taxane chemotherapy regimens rather than additional AR targeted therapies^17^. However, Belderbos et al*.* found that AR-V7 status in CTCs had no prognostic value in patients with mCRPC, who progressed after docetaxel and/or enzalutamide or abiraterone and were started on CBZ as the next line of treatment^18^. However, the authors noted that further prospective trials are needed^18^, and this current study adds to that evidence base.

Since AR-V7 is most sensitively detected in CTC samples^19^, we collected and analyzed CTCs using AdnaTest kit. According to recent findings, the number of CTCs must be considered when describing the relationship between AR-V7 status and prognosis in CTCs^20^. Even though AdnaTest measured CTC to be negative, CellSearch detected 69.5% of CTCs^20^, implying that AdnaTest was less sentitive than CellSearch. However, in this study, we used AdnaTest as a “Point Of Care Test,” focusing on the simplicity and feasibility of the CTC analysis. In this study, we demonstrated that AR-V7 in CTCs could be detected in any laboratory without the use of expensive equipment, and the results can be applied in the context of precision medicine. It was also considered that AdnaTest would be useful in clinical practice as a POCT tool that could easily evaluate AR-V7 status even if the CTC detection sensitivity was slightly inferior. Thus, we used the AdnaTest to perform the following three-group analysis: CTC-negative/AR-V7-negative (29%), CTC-positive/AR-V7-negative (54%), and CTC-positive/AR-V7-positive (17%) (Table 3). Antonarakis et al. Used the same three prognostic categories (26%, 56%, and 18%, respectively) after treatment with abiraterone or enzalutamide^9^. Although several studies investigated two groups, AR-V7-positive and AR-V7-negative, in which CTC could be detected, it was also interesting to see if there was any difference in efficacy from the CTC negative group, as reported by Antonarakis et al. and Cattrini et al.^9,21^. CTC detection is the most important prognostic factor for drug efficacy in mCRPC^22^. In our study, we found no significant difference in survival between the presence and absence of CTC detection (Fig. 3). One reason might be the insufficient number of cases and the lack of test power. Secondly, AdnaTest is considered to be less sensitive than CellSearch, so using CellSearch might have resulted in a significant reduction in the number of CTC negatives. Third, Sharp et al. reported that 63% (10/16) of the AdnaTest-detected patients who were CTC-negative had detectable AR-V7 protein expression in their matched mCRPC biopsy^20^. Thus, it is possible that the CRPC tissue itself is AR-V7 positive below the CTC detection limit. Furthermore, such cases may have a poor prognosis.

We propose that the effect of CBZ on bone metastases is also independent of CTCs and AR-V7 status (Fig. 4a). Furthermore, the effect of CBZ on bone metastases—a decrease in BSI as measured by bone scintigraphy, could be associated with prolongation of survival (Fig. 4b). The use of bone scintigraphy to measure BSI is an objective parameter to evaluate bone metastases and is a useful tool for the diagnosis and evaluation of metastatic prostate cancer in clinical practice.

In a retrospective study, Reza et al*.* reported that reduction of BSI (ΔBSI) after abiraterone administration correlated with survival time in patients with mCRPC^23^. A retrospective study by Anand et al*.* also showed that ΔBSI after enzalutamide administration was correlated with survival in patients with mCRPC and bone metastases^24^. However, a subsequent retrospective study by Miyoshi et al. reported no such correlation between ΔBSI and OS after 16 weeks of CBZ administration^25^. Although the findings of Miyoshi et al*.* differ from our findings, there are differences between the studies: prospective versus retrospective study design; slightly larger patient population (48 versus 32 patients); early versus late treatment line with CBZ in mCRPC; and a higher rate of visceral metastases. While there were not many cases of visceral metastases in this study, over 90% of patients had bone metastases; it is, therefore, theoretically convincing that the effect of CBZ on bone metastases contributed to the prolongation of survival.

There were no new safety concerns identified in this cohort of Japanese patients with mCRPC receiving prophylactic pegfilgrastim, suggesting that tolerability to CBZ in Japanese patients is comparable to that of mCRPC patients in Western countries.

This study has a number of limitations as well. First, because CBZ was most frequently administered in patients with relatively advanced stages of mCRPC, it was challenging to evaluate all patients until the end of the study period (seven patients withdrew prematurely). Second, the false positive rate due to benign bone degeneration and medication-related osteonecrosis of the jaw might have affected the analyses in patients with a low percentage of bone metastases (BSI < 1%). Third, and most important, we used AdnaTest exclusively for CTC collection and analysis procedures. If we had used a highly sensitive device capable of counting CTCs, such as CellSearch, the number of CTC-negative groups would have significantly decreased. It may have also contributed to a higher survival rate in the CTC-negative group. Lastly, the full-length andogen receptor (AR-FL) could have been analyzed alongside AR-V7 as an additional associated marker in mCRPC^21^. Additionally, other androgen receptor variants, such as AR-V1, AR-V3, and AR-V9, could have been analyzed for completeness^26^.

## Conclusions

The findings of this Japanese cohort study confirm previously reported outcomes for CBZ in patients with mCRPC from Western countries who were previously treated with docetaxel. The detection of AR-V7 in CTCs was not associated with CBZ resistance. Findings also suggest that the BSI-reducing response using bone scintigraphy and the PSA-reducing response during the initial stages of CBZ administration could be important predictors of CBZ efficacy on OS rate. CTC-based AR-V7 detection has the potential to be used as a treatment selection biomarker in patients with mCRPC. AdnaTest would be useful in clinical practice as a POCT tool for evaluating AR-V7 status.

## Materials and methods

### Patients

Patients with mCRPC who had progressed on docetaxel were recruited from Juntendo University Hospitals (between February 2017 and August 2020) for phase 2, a non-randomized, open-label, single-arm, estimation-based study. The baseline AR-V7 status of CTCs was determined, and all patients received CBZ irrespective of AR-V7 status. Patients received 20 mg/m^2^ of CBZ every 3–4 weeks until disease progression or the occurrence of unacceptable toxicity. To reduce the incidence of severe febrile neutropenia, Granulocyte Colony Stimulating Factor (G-CSF; longer-acting pegylated GCSF [pegfilgrastim]) was administered prophylactically to all patients beginning with the first cycle of CBZ.

This study was approved by the Institutional Review Board of Juntendo University Hospital (admission number: 14–052), and all experiments were carried out in accordance with approved guidelines. All participants provided written informed consent.

### CTC analyses

The AdnaTest (QIAGEN, Germany) was used to detect CTCs in accordance with the manufacturer’s protocol: 5 mL of blood was collected in EDTA-3 K tubes, RNA was extracted using antibody-conjugated magnetic beads (AdnaTest ProstateCancerSelect), and mRNA was then extracted using AdnaTest ProstateCancerDetect^4,9,27^. The extracted mRNA was subjected to reverse transcription using the Sensiscript Reverse Transcriptase Kit (QIAGEN). Reverse transcription polymerase chain reaction (RT-PCR) was used to detect PSA, AR-V7, and AR in CTCs. PSA and AR were amplified using the AdnaTest PrimerMix ProstateDetect (PCR conditions for PSA: 95 °C for 15 min, 42 cycles of 30 s at 94 °C, 61 °C for 30 s, 72 °C for 30 s, followed by 10 min of extension, and PCR conditions for AR: 95 °C for 15 min, 35 cycles of 30 s at 94 °C, 60 °C for 30 s, 72 °C for 60 s, followed by 10 min of extension). These experiments confirmed that all samples were positive for PSA. Thus, we concluded that PSA positivity is a common denominator in this study and defined successful CTC detection as positive PSA expression. The primer set and PCR conditions for AR-V7 RT-PCR are as follows: AR-V7 primer set designed to yield 125-bp AR-V7-specific band—5′-CCATCTTGTCGTCTTCGGAAATGTTA-3′ and 5′-TTTGAATGAGGCAAGTCAGCCTTTCT-3′ (PCR conditions for AR-V7: 95 °C for 5 min, 39 cycles at 95 °C for 10 s, 58 °C for 30 s, 72 °C for 30 s, followed by 10 min of extension). Amplified PCR products were electrophoresed and visualized by the DNA 1 K Experion™ automated electrophoresis system (Bio-Rad, CA, USA). To evaluate gene expression, the fluorescence intensity scale was set to “scale to local” (default setting), and any visible bands with detectable peaks under these conditions were considered positive.

### Study endpoints

The primary end point was PSA response rate (PSA-RR), and the primary objective was to compare PSA-RR between CTC-negative, AR-V7-positive, and AR-V7-negative (CTC-positive) cohorts. PSA-RR was defined as a ≥ 30% and ≥ 50% decline in PSA level from baseline to 12 weeks of CBZ treatment, or earlier if treatment was discontinued. Secondary end points included overall survival in patients with or without PSA decline 3 months following CBZ treatments, with or without CTCs and AR-V7 status at baseline, and the relationship with or without decline of Bone Scan Index (BSI) by bone scintigraphy. The safety of CBZ in Japanese patients with mCRPC was assessed using CTCAE 4.0 protocol.

### Statistical analysis

The Kruskal–Wallis test was used for statistical analysis of patient background. Survival was analyzed using Cox regression models and visualized in Kaplan–Meier plots, and OS curves were generated using the Kaplan–Meier method. The PSA-RR (≥ 50% and ≥ 30% decline in PSA from baseline PSA following chemotherapy) was compared for three groups: CTC-negative/AR-V7-negative; CTC-positive/AR-V7-negative; and CTC-positive/AR-positive; and analyzed using the chi-square or Fisher’s exact tests. Statistical differences between the three groups were compared using the log-rank test. 

## Supplementary Information

Supplementary Information 1.

Supplementary Information 2.

## Data Availability

The datasets generated and analyzed during this study are available in the NBDC (National Bioscience Database Center) Human Database repository, [https://humandbs.biosciencedbc.jp/en/ Research ID : hum0354].
