# Supplementary material for: Efficacy of cabazitaxel and androgen splicing variant-7 status in circulating tumor cells in Asian patients with metastatic castration-resistant prostate cancer
Source: Sci Rep. 2022 Oct 26;12:18016. doi: 10.1038/s41598-022-22854-1 (PMC9606294; doi:10.1038/s41598-022-22854-1)
Supplement: Supplementary file 1 — Supplementary Information 1. [file 41598_2022_22854_MOESM1_ESM.pptx]

## Slide 1
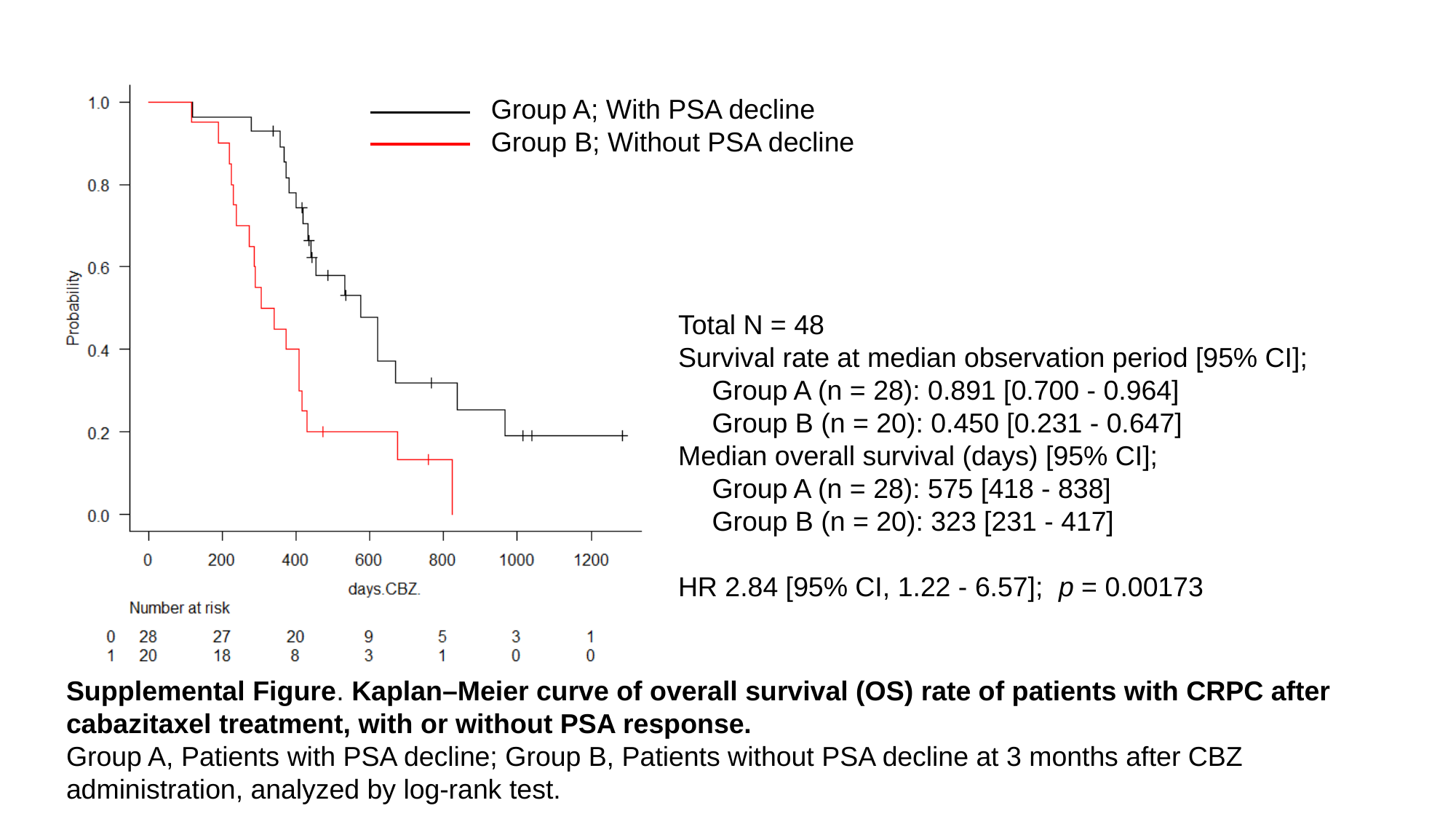

Group A; With PSA decline
Group B; Without PSA decline
Total N = 48
Survival rate at median observation period [95% CI];
　Group A (n = 28): 0.891 [0.700 - 0.964]
　Group B (n = 20): 0.450 [0.231 - 0.647]
Median overall survival (days) [95% CI];
　Group A (n = 28): 575 [418 - 838]
　Group B (n = 20): 323 [231 - 417]
HR 2.84 [95% CI, 1.22 - 6.57]; p = 0.00173
Supplemental Figure. Kaplan–Meier curve of overall survival (OS) rate of patients with CRPC after cabazitaxel treatment, with or without PSA response.
Group A, Patients with PSA decline; Group B, Patients without PSA decline at 3 months after CBZ administration, analyzed by log-rank test.
