# Supplementary material for: Efficacy of cabazitaxel and androgen splicing variant-7 status in circulating tumor cells in Asian patients with metastatic castration-resistant prostate cancer
Source: Sci Rep. 2022 Oct 26;12:18016. doi: 10.1038/s41598-022-22854-1 (PMC9606294; doi:10.1038/s41598-022-22854-1)
Supplement: Supplementary file 2 — Supplementary Information 2. [file 41598_2022_22854_MOESM2_ESM.pptx]

## Slide 1
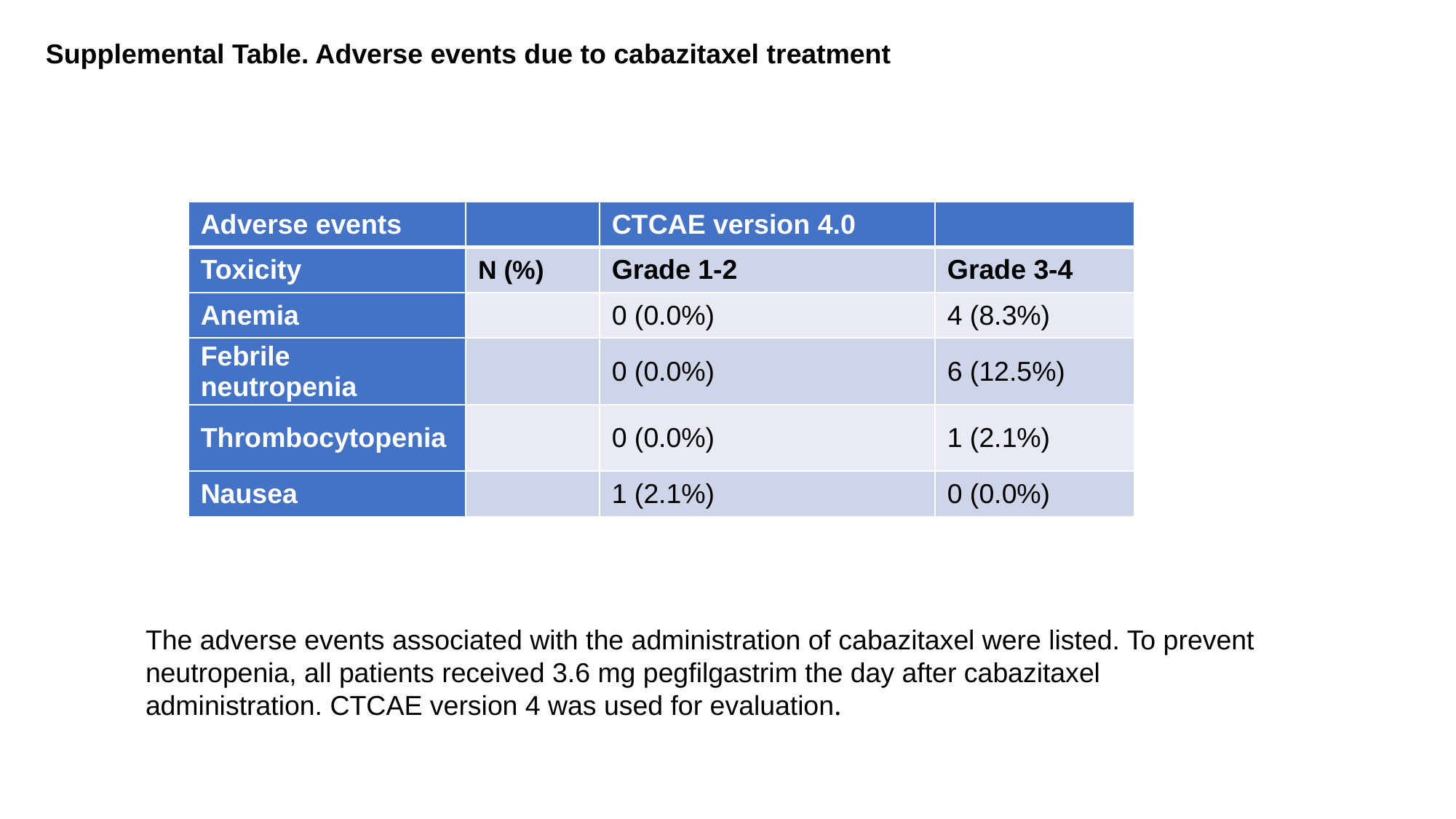

Supplemental Table. Adverse events due to cabazitaxel treatment
| Adverse events | | CTCAE version 4.0 | |
| --- | --- | --- | --- |
| Toxicity | N (%) | Grade 1-2 | Grade 3-4 |
| Anemia | | 0 (0.0%) | 4 (8.3%) |
| Febrile neutropenia | | 0 (0.0%) | 6 (12.5%) |
| Thrombocytopenia | | 0 (0.0%) | 1 (2.1%) |
| Nausea | | 1 (2.1%) | 0 (0.0%) |
The adverse events associated with the administration of cabazitaxel were listed. To prevent neutropenia, all patients received 3.6 mg pegfilgastrim the day after cabazitaxel administration. CTCAE version 4 was used for evaluation.
